# Stalks and roots are the main battlefield for the coevolution between maize and *Fusarium verticillioides*

**DOI:** 10.3389/fpls.2024.1461896

**Published:** 2024-10-16

**Authors:** Hao Xiong, Xiaobin Xing, Muyuan Liu, Zhaoyu Zhang, Qingjun Wang, Xuemei Zhang, Xiangjian Gou, Yanli Lu, Xuanjun Feng

**Affiliations:** ^1^ Maize Research Institute, Sichuan Agricultural University, Sichuan, Chengdu, China; ^2^ State Key Laboratory of Crop Gene Exploration and Utilization in Southwest China, Sichuan, Chengdu, China; ^3^ National Key Laboratory of Crop Genetic Improvement, Huazhong Agricultural University, Wuhan, Hubei, China

**Keywords:** pathogen-host interaction, coevolution, maize, fusarium, ear rot

## Abstract

*Fusarium* species are the dominant cause of maize ear rot, but they also inflict serious damage to the roots and stalks. Theoretically, the organ where the host interacts with the pathogen most frequently should exhibit the highest degree of symptom-genotype correlation. Because that symptom-genotype correlation is an indicator reflecting the degree of coevolution between pathogen and its hosts. We wonder which organ is the main battlefield for the antagonism between maize and *Fusarium.* For this purpose, 43 isolates of *Fusarium* were isolated from infected maize ears. *Fusarium verticillioides* and *F. graminearum* are the two dominant pathogens, accounting for 44% and 30%, respectively. Furthermore, 14 elite maize inbreds were exposed to 43 *Fusarium* isolates and the symptoms of ear rot, stalk rot and root rot were investigated. In general, symptoms caused by *F. graminearum* were significantly more severe than those caused by other *Fusarium* species. Surprisingly, the genotype of *F. verticillioides* showed a strong correlation with stalk and root rot, but not with ear rot. Accordingly, our study may provide the first evidence that the stalk and root of maize, rather than the ear, is the main battlefield for the coevolution between maize and *F. verticillioides*.

## 1 Introduction

Maize (*Zea mays* L.) is a crucial global source of food, feed, and energy. In recent decades, the demand for increased maize yields has been a primary driver behind the ongoing development of commercial varieties. However, as maize yield gains have gradually slowed, the rise of destructive diseases has become the leading factor propelling the renewal of commercial varieties (Ristaino et al., 2021). Therefore, understanding the interaction between pathogens and maize is important for maize production.

Maize ear rot is a destructive disease, mainly caused by *Fusarium* species, poses a significant threat to maize production and quality (Duan et al., 2016). *Fusarium* species overwinter in soil, seeds, and plant debris (Xia et al., 2022). They spread to maize grains through the roots, insects, and the air (Xia et al., 2022; Omotayo and Babalola, 2023). They also cause root and stalk rot, affecting overall plant health and yield (María I et al., 2017). In addition, *Fusarium* species can produce harmful mycotoxins such as deoxynivalenol (DONs), fumonisins (FBs) or Zearalenone (ZENs), which pose a risk to animal and human health if consumed in excessive amounts (María I et al., 2017). In China, significant yield losses and mycotoxin contamination problems have been associated with *Fusarium* species (Duan et al., 2016; Qin et al., 2020).

The pathogen-host-driven natural selection contributes to shape the genetic and phenotypic diversities and results in coevolution of both organisms (Sironi et al., 2015). Pathogens have a negative effect on host fitness, favoring selection for enhanced defense mechanisms in the affected hosts. Conversely, host defenses are detrimental to the pathogen, leading to selection for novel attack mechanisms. When the interaction persists over time, the ongoing cycles of antagonism can generate highly specific host-pathogen interaction patterns, which are intrinsically determined by specific genotype interaction and are manifested as lineage-specific invasion (Schneider et al., 2015; Delaux and Schornack, 2021). Symptom-genotype correlation, the manifestation of specific genotype interaction, is a simple and feasible indicator reflecting the degree of coevolution between pathogen and its hosts. Theoretically, the organ in which the host interacts with the pathogen most frequently should exhibit the highest degree of symptom-genotype correlation. Given the extensive research conducted on the interaction between *Fusarium* and maize ears, we subjectively think that ears are the main battlefield for the antagonism between maize and *Fusarium* species. However, our studies unexpectedly revealed that the genotype of *F. verticillioides* was more closely related to its pathogenicity to maize stalks and roots than to maize ears. Prior to this study, there was no direct evidence that the localized interactions between *Fusarium* species and maize were the main factor driving its coevolution. Our study may provide the first evidence that the stalk and root of maize, rather than the ear, is the main battlefield for the coevolution between maize and *F. verticillioides*.

## 2 Results

### 2.1 The isolation frequency of *F. verticillioides* from maize ear rot is the highest in China

**Figure 1:**
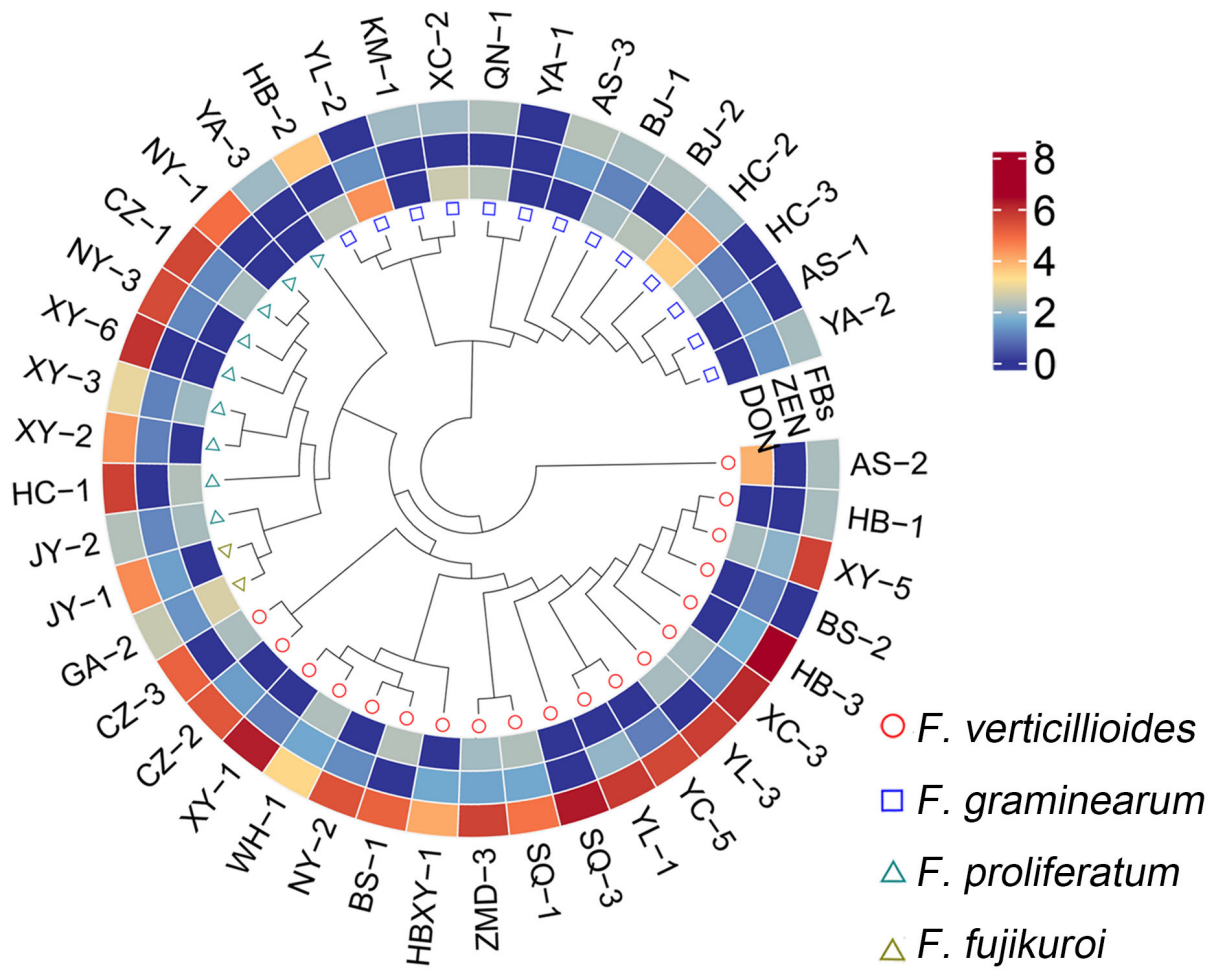
Topological phylogenetic tree of 43 *Fusarium* isolates and toxigenic capacity. The genetic distance matrix of 43 *Fusarium* isolates is showed as topological phylogenetic tree. The detail information was shown in **Supplementary Table S4**. The out layer heatmap represents the toxigenic capacity of different isolates grown in maize medium. The data used in heatmap are modified from **Supplementary Table S1** by Log10 conversion. If the contents of FBs, T2 and DON were less than 100 μg/kg and of ZEN less than 10 μg/kg, the result was inaccurate and recorded as zero. FBs, Fumonisin; ZEN, zearalenone; DON, deoxynivalenol.

Different isolates of *Fusarium* spp. were isolated from infected maize ears from 20 cornfields in 7 provinces in China (**Supplementary Table S1**). Forty-three isolates were isolated and grouped into 4 categories, *F. verticillioides* (44%), *F. graminearum* (30%), *F. proliferatum* (21%), and *F. fujikuroi* (5%) (**Supplementary Table S1**), which is consistent with that *F. verticillioides* was previously reported as the dominant ear rot pathogen (Logrieco et al., 2002; Folcher et al., 2009; Lanubile et al., 2017; Feng et al., 2022). Fumonisin production was high for most *F. verticillioides* and *F. proliferatum* isolates but low for *F. graminearum* isolates when grown on maize medium, and no T2 toxin was detected from any isolates (**Figure 1**; **Supplementary Table S1**).

### 2.2 The symptoms caused by *F. graminearum* are significantly more severe than that caused by *F. verticillioides and F. proliferatum*

**Figure 2:**
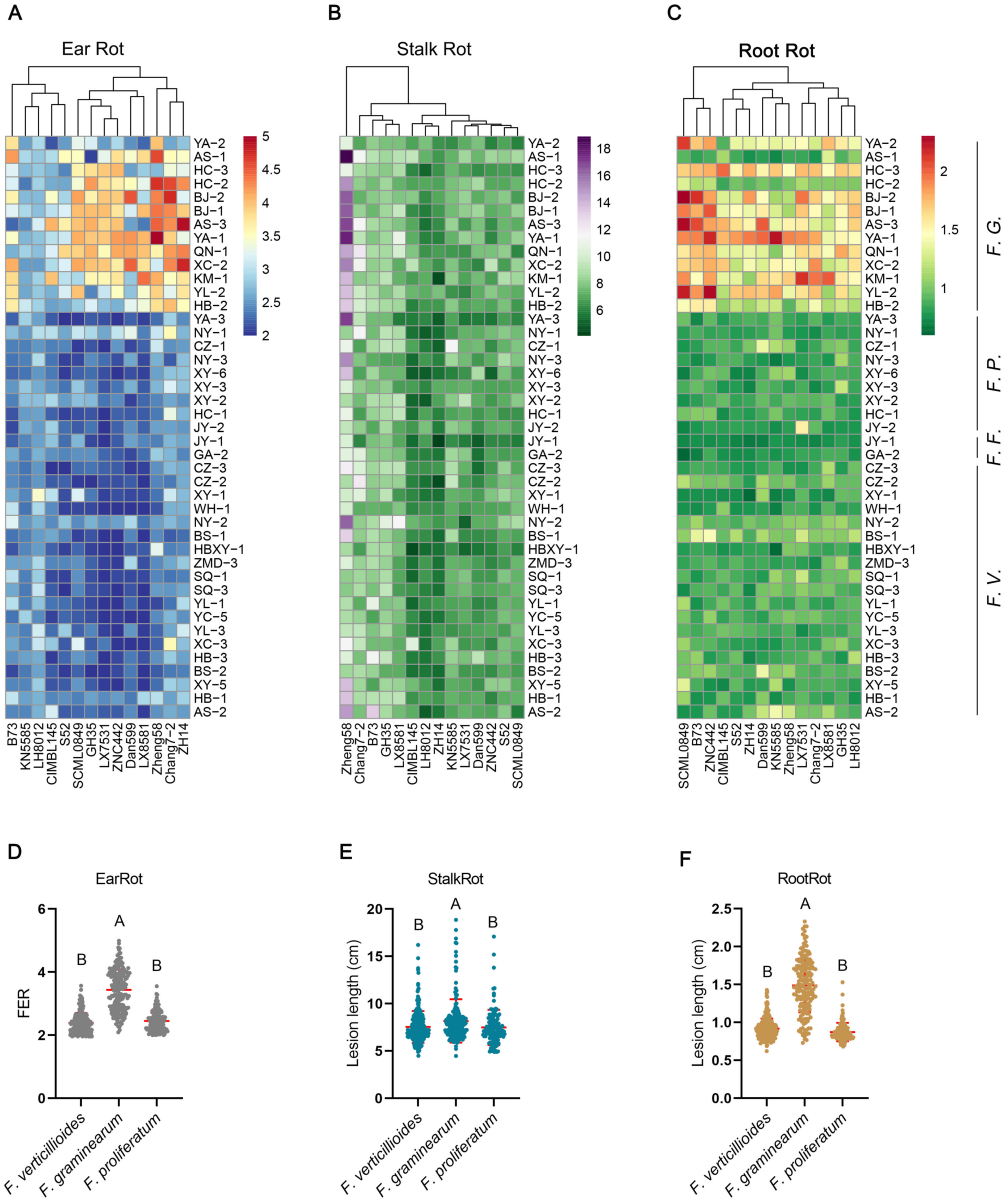
Severity of ear rot, stalk rot, and root rot of different pathogen-host pairwise interactions. **(A-C)** Severity of symptom for each pathogen-host pairwise was shown as heatmap. **(D-F)** Scatter plot of symptom severity caused by *F. verticillioides (F.V.), F. graminearum* (*F.G.*) and *F. proliferatum (F.P.). F.F.* is the abbreviation for *F. fujikuroi.* One point represents one pathogen-host interaction pairwise. The different capital letters above the scatter indicate that the difference between groups is significant at the level of *p*-value less than 0.01 using ANOVA analysis.

The pathogenicity of 43 isolates was tested using 14 elite maize inbred lines. All 43 isolates could infect maize ears, stalks and roots, and pathogen-host interactions showed a wide range of symptom severity (**Supplementary Table S2**; **Figure 2**). The pathogenicity of *F. graminearum* was remarkably higher than that of *F. verticillioides* and *F. proliferatum*, and was comparable between *F. verticillioides and F. proliferatum* (**Supplementary Table S2**; **Figures 2D-F**). KN5585 and LH8012 exhibited strong resistance to ear rot caused by *F. graminearum* (**Figure 2A**). LX7531, ZNC442 and LX8581 displayed good resistance to ear rot caused by *F. verticillioides* (**Figure 2A**). CIMBL145, LH8012, and ZH14 showed effective resistance to most *Fusarium*-caused stalk rot, whereas Zheng58 was susceptible to most *Fusarium*-caused stalk rot (**Figure 2B**).

### 2.3 Stalks and roots are the main battlefield for the coevolution between maize and *F. verticillioides*

**Figure 3:**
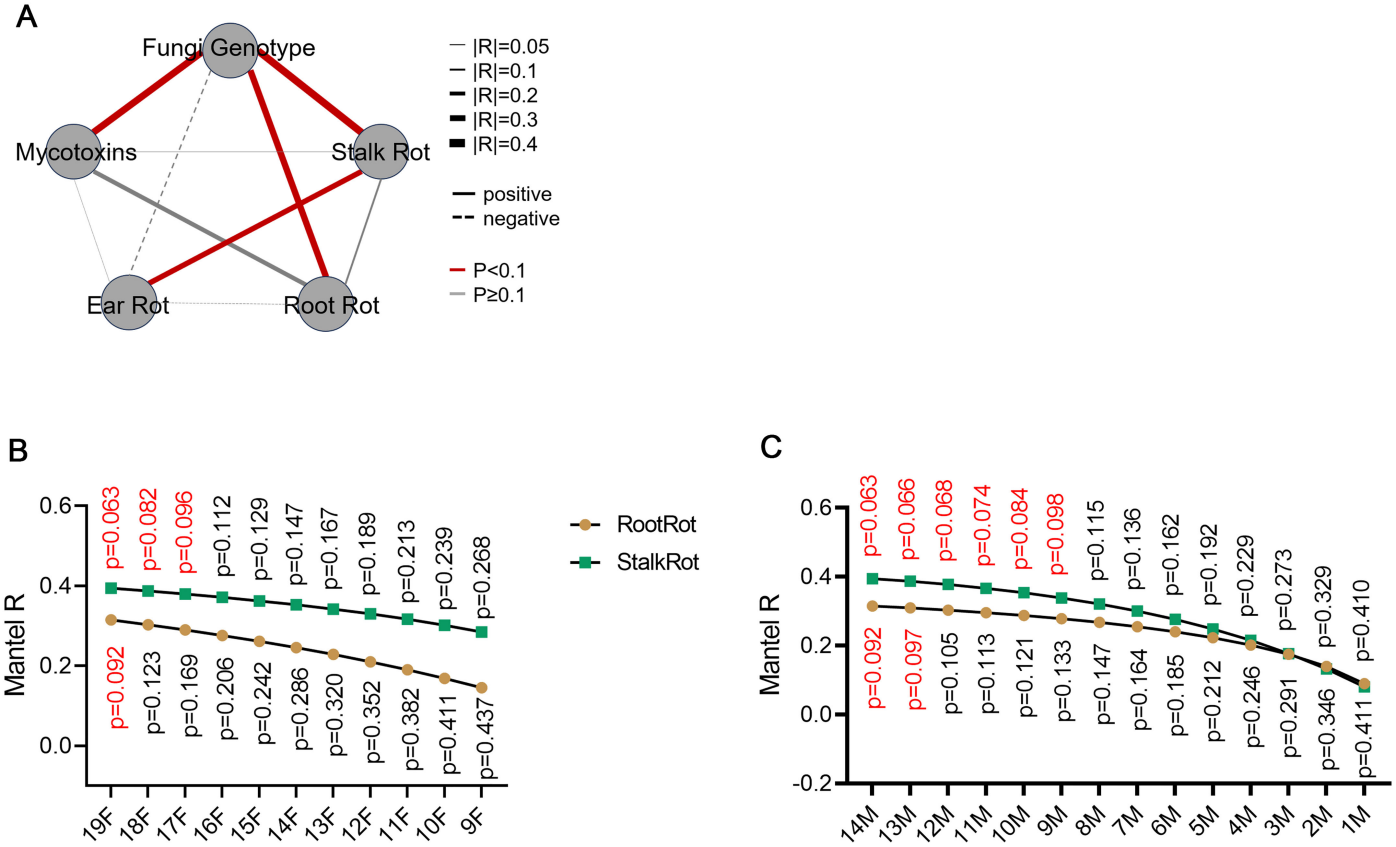
Stalks and roots are the main battlefield for the coevolution between maize and *F. verticillioides.* **(A)** Correlation between different traits of *F. verticillioides*. The correlation is considered statistically significant when the *p*-value is less than 0.1. The detail information of each trait was shown in **Supplementary Tables S4-8**. Mantel test was used to calculate the correlation and *p*-value between different traits. **(B)** The effect of pathogen diversity on the symptom-pathogenic genotype correlation was tested by retaining different numbers of *F. verticillioides* isolates. **(C)** The effect of maize diversity on the symptom-pathogenic genotype correlation was tested by retaining different numbers of inbred maize lines. For **(B, C)**, The distance matrix of different traits was used to calculate correlations using the Mantel test with 999 permutations.

The relationship between fungal genotype, toxigenic capacity, and pathogenicity was further investigated. The correlation between the genotype of *F. verticillioides* and the symptom of stalk and root rot, but not ear rot, was notable (**Figure 3A**; **Supplementary Tables S3-8**). This implies that the coevolution of *F. verticillioides* and maize is mainly existed in the root and stalk, rather than the ear. The number of isolates of *F. graminearum* and *F. proliferatum* observed in this study was relatively low, which may explain why no significant correlation was found between the genotypes of these two pathogens and the different symptoms observed (**Supplementary Tables S3-8**). To test the effect of racial diversity on the genotype-symptom correlation, different numbers of *F. verticillioides* were progressively removed. With the decrease of racial diversity, the genotype-symptom correlation coefficient decreased, and the corresponding *p* value increased (**Figure 3B**). When the number of isolates was less than 17, the symptom-genotype correlation was no longer significant for both root and stalk rot (**Figure 3B**). To test the effect of maize diversity on the genotype-symptom correlation, different numbers of maize inbred lines were progressively removed. With the decrease of maize diversity, the genotype-symptom correlation coefficient decreased, and the corresponding *p* value increased (**Figure 3C**). These results indicated that in order to ascertain the correlation between genotype and symptoms with sufficient clarity, it is necessary to ensure that the pathogens and hosts under consideration exhibit sufficient diversity. An expansion of the pathogen-host interacting population results in a heightened correlation between genotype and symptoms.

The toxigenic capacity is correlated well with fungal genotype in *F. verticillioides* and *F. proliferatum*, but not in *F. graminearum* (**Figure 3A**; **Supplementary Table S3**). The toxigenic capacity is not correlated with the symptom of ear rot, stalk rot, or root rot, indicating that the toxigenic capacity may not be a dominant determinate of pathogenicity.

## 3 Discussion

*F. verticillioides* and *F. graminearum* are the most widely reported causes of maize ear rot (Logrieco et al., 2002; Folcher et al., 2009; Gai et al., 2017; Lanubile et al., 2017; Feng et al., 2022). They also cause stalk, root and sheath rots (Duan et al., 2016; Lanubile et al., 2017; Wang et al., 2021). In this study, 44% of isolates are *F. verticillioides*, 30% are *F. graminearum* (30%), and 21% are *F. proliferatum* (21%). It is consistent with previous reports that *F. verticillioides* and *F. graminearum* are the dominant maize ear rot pathogens.

The distributions and pathogenicity of *F. verticillioides* and *F. graminearum* vary greatly in different environmental and climatic conditions (Ezrari et al., 2021; Liu et al., 2022). Although both *F. verticillioides* and *F. graminearum* can cause ear, stalk, and root rot in maize, there seems to be no studies that systematically compare the pathogenicity of the two pathogens at the population level. It was reported that the optimum temperature for growth of *F. verticillioides* is about 30°C, while cool growing conditions (20°C) promote the growth of *F. graminearum* (Samapundo et al., 2005; Hay et al., 2021; Ding et al., 2024). In this study, the field temperatures after inoculation were usually between 25 and 35°C, which may be more suitable for the growth of *F. verticillioides*. However, the results clearly showed that the severity of disease caused by *F. graminearum* to 14 elite maize inbred lines was significantly greater than that of *F. verticillioides*. This indicates that the pathogenicity of *F. graminearum* to maize is generally stronger than that of *F. verticillioides*.

*F. verticillioides* can survive in soil, seeds, and plant debris for extended periods. It is primarily a soil- and seed-borne fungus that moves from the root upward to the maize stalks and ears, causing stalk and ear rot (Omotayo and Babalola, 2023). It can also survive the winter as viable spores and is spread by airborne or insect vectors and rainfall (Omotayo and Babalola, 2023). The widespread existence of *F. verticillioides* in the soil made it necessary for maize roots to cope with the invasion of *F. verticillioides* at all times. Given the important economic attributes of maize ear, ear rot has received more attention than root and stalk rot. Thus, we subjectively think that ears may be the main battlefield for the antagonism between maize and *Fusarium* species before this study. To the best of our knowledge, there is currently no evidence can indicate the main organ in which the coevolution of maize and *F. verticillioides* has occurred. The organ where the pathogen frequently interacts with the host is expected to show the highest degree of symptom-genotype correlation (Schneider et al., 2015; Sironi et al., 2015; Delaux and Schornack, 2021). This correlation serves as a simple and feasible indicator of coevolution, as it is intrinsically shaped by the genotype interactions between pathogen and its hosts (Schneider et al., 2015; Sironi et al., 2015; Delaux and Schornack, 2021). Our findings indicate that the genotype of *F. verticillioides* is strongly associated with stalk and root rot, but not with ear rot. Consequently, this study may provide the first evidence that the stalk and root of maize, rather than the ear, is the main battlefield for the antagonism and coevolution between maize and *F. verticillioides*. The characteristics of the soil- and seed-borne form of *F. verticillioides* may explain why the antagonism and coevolution occurred mainly in the maize stalk and root.

Theoretically, a stronger long-term antagonism may result in a higher symptom-genotype correlation, while an increasing size of the pathogen-host interacting population may enhance this correlation (Schneider et al., 2015; Sironi et al., 2015; Delaux and Schornack, 2021). However, the specific size of the pathogen-host interacting population in which this correlation can be remarkably observed remains unclear. In previous studies, the correlation between genotype and symptoms was usually low or not statistically significant at all (Gai et al., 2017; Li et al., 2019; Han et al., 2020; Dong et al., 2022; Feng et al., 2022; Liu et al., 2024)(**Supplementary Table S9**). This may be due to the fact that the pathogen-host interacting populations were constructed with only one pathogen or host. In line with this, a decrease in the diversity of *F. verticillioides* or maize will decrease the genotype-symptom correlation coefficient and increase the corresponding *p*-value. In order to ascertain the correlation between genotype and symptoms with sufficient clarity, it is necessary to ensure that the pathogens and hosts under consideration exhibit sufficient diversity. Here, we suggested that the size of the pathogen-host interacting population is larger than a 20 × 15 matrix will be better. Our results showed that symptom-genotype correlation is a simple and feasible indicator of the degree of coevolution between pathogens and hosts, when the size of the pathogen-host interacting population is appropriate.

## 4 Materials and methods

### 4.1 Isolation of *Fusarium* spp.

Infected maize ears were collected from 20 locations in 7 provinces in southwest China. Asymptomatic kernels adjacent to the infected aera were surface sterilized with 75% ethanol for 5 min and incubated on potato dextrose agar (PDA) medium at 25°C. Pure culture of pathogen was established by single spore isolation. Three housekeeping genes, the translation elongation factor *EF1α*, the largest (*RPB1*) and second largest (*RPB2*) subunits of RNA polymerase, were amplified by polymerase chain reaction (PCR) for resequencing. Sequences were aligned in the Fusarium database (https://fusarium.mycobank.org/) for species identification. Primers are listed in **Supplementary Table S10**.

### 4.2 Analysis of genetic distance

The sequences of three housekeeping genes (*EF1α, RPB1, and RPB2*) were used for phylogenetic analysis and computing of pairwise genetic distance. Analyses were conducted using the Maximum Composite Likelihood model in MEGA7 (Kumar et al., 2016). Pairwise genetic distance was further used for correlation analysis with other phenotypes.

### 4.3 Determination of the content of four mycotoxins

As previous reported, *Fusarium* species were cultured in solid maize sand medium at 28 °C in the dark for 5 days. The contents of fumonisins (FBs), deoxynivalenol (DON), T-2, and zearalenone (ZEN) in the medium were determined with FD-600 (Femdetection, China) using immunofluorescence-based rapid quantitative test strips (Feng et al., 2022).

### 4.4 Artificial inoculation and symptom investigation of ear, stalk and root rots

To investigate the severity of ear and stalk rots, 14 elite inbred maize lines were planted in 2022 in Xishuangbanna (21° 53′ N, 100° 59′ E) and in 2023 in Chongzhou, China (30° 33′ N, 103° 39′ E). Seedlings were planted with 3.5 m single rows and 0.8 m row widths. Approximately 60 days after planting, a hole about 1 cm deep was made in the middle of the second stalk node using a 1 mm diameter electric drill. The hole was inoculated with 200 μL of spore suspension (5 × 10^6^ spores/mL) and the lesion length was measured when the seed was mature. Fourteen days after silking for each inbred maize line, 200 μL of spore suspension (5 × 10^6^ spores/mL) was inoculated into the ears using the side-needle syringe method. The severity of ear rot was assessed as in our previous report, based on the agricultural industry standard of the People’s Republic of China (NY/T1248.8-2016) (Feng et al., 2022). For each pathogen-host interaction, 20 ears or stalks from each replicate and two replicates at each location were investigated.

To assess the severity of root rot, healthy seeds were soaked in water overnight and incubated on wet seed germination paper at 25°C for about 2 days in the dark. Seeds with uniform growth were selected and transferred to fresh wet seed germination paper in a 13 × 13 cm Petri dish for 3 days of continuous growth. A wound was created by needle at the root position at about 4 cm from the seed, and then a hyphae-covered clump of PDA (0.3 cm diameter) was touched to induce root rot. The seeds were covered with wet germination paper to keep them moist and to ensure that the roots grew straight. About 4 days after inoculation, the lesion length was measured to reflect the severity of root rot. The best linear unbiased prediction (BLUP) values for each trait from two years were used to calculate symptoms distance matrix. Representative photos illustrating the inoculation site and symptoms of ear rot, stem rot, and root rot are presented in **Supplementary Figure S1**.

### 4.5 Correlation analysis between different traits

The vegdist function of the vegan package (https://CRAN.R-project.org/package=vegan) (Dixon, 2003) was used to construct the distance matrix of different isolates for the toxin production, the symptoms of ear, stalk and root rot. The mantel function was then used to calculate the correlation and *p*-value between different traits. To test the effect of pathogen and host diversity on the correlation, different numbers of pathogens or hosts were removed and the corresponding correlation and *p* value was calculated. For example, if three of the fourteen inbred maize lines were removed, 364 combinations and 364 correlation and *p*-values would be generated. Therefore, the correlation and *p* value would be calculated from the mean of the 364 values when the host diversity was eleven.

## Data Availability

The datasets presented in this study can be found in online repositories. The names of the repository/repositories and accession number(s) can be found in the article/**Supplementary Material**.
