# Supplementary material for: Stalks and roots are the main battlefield for the coevolution between maize and Fusarium verticillioides
Source: Front Plant Sci. 2024 Oct 16;15:1461896. doi: 10.3389/fpls.2024.1461896 (PMC11521819; doi:10.3389/fpls.2024.1461896)
Supplement: Supplementary file 12 [file Table11.docx]

Supplementary Material

## Supplementary Figures


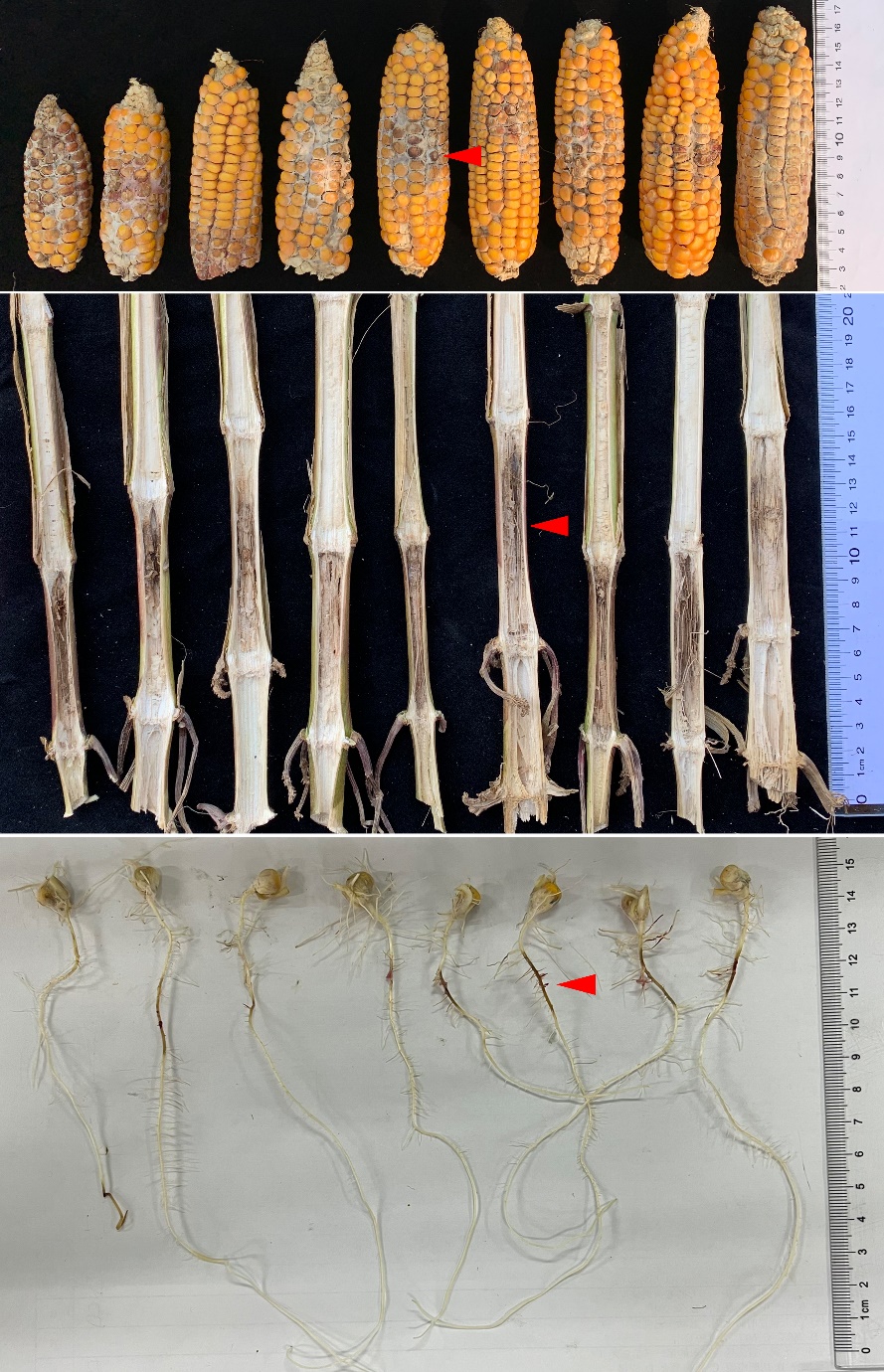


**Supplementary Figure 1.** The representative photos to display the inoculation site and symptoms of ear rot, stem rot and root rot. The red arrows indicate the inoculation site and symptoms.

**Supplementary Tables**

**Table S1. Information of different *Fusarium* isolates.**

**Table S2. General descriptive statistics of *F. verticillioides*, *F. graminearum* and *F. proliferatum* induced symptoms.**

**Table S3. Correlation between different traits.**

**Table S4. The genotypic distance matrix of different *Fusarium* isolates.**

**Table S5. Data of mycotoxins used for distance matrix calculation.**

**Table S6. Severity of ear rot of different pathogen-host interactions.**

**Table S7. Severity of stalk rot of different pathogen-host interactions.**

**Table S8. Severity of root rot of different pathogen-host interactions.**

**Table S9. Symptom-genotype correlation in previous reports.**

**Table S10. Primers used in this study.**
